# Combinations of Physical Activity, Sedentary Behavior, and Sleep Duration and Their Associations With Physical, Psychological, and Educational Outcomes in Children and Adolescents: A Systematic Review

**DOI:** 10.1093/aje/kwac212

**Published:** 2022-12-14

**Authors:** Katrina Wilhite, Bridget Booker, Bo-Huei Huang, Devan Antczak, Lucy Corbett, Philip Parker, Michael Noetel, Chris Rissel, Chris Lonsdale, Borja del Pozo Cruz, Taren Sanders

**Keywords:** adolescents, children, physical activity, sedentary behavior, sleep

## Abstract

We conducted a systematic review to evaluate combinations of physical activity, sedentary behavior, and sleep duration (defined as “movement behaviors”) and their associations with physical, psychological, and educational outcomes in children and adolescents. MEDLINE, CINAHL, PsychInfo, SPORTDiscus, PubMed, EMBASE, and ERIC were searched in June 2020. Included studies needed to 1) quantitatively analyze the association of 2 or more movement behaviors with an outcome, 2) analyze a population between 5 and 17 years of age, and 3) include at least an English abstract. We included 141 studies. Most studies included the combination of physical activity and sedentary behavior in their analyses. Sleep was studied less frequently. In combination, a high level of physical activity and a low level of sedentary behavior were associated with the best physical health, psychological health, and education-related outcomes. Sleep was often included in the combination that was associated with the most favorable outcomes. Sedentary behavior had a stronger influence in adolescents than in children and tended to be associated more negatively with outcomes when it was defined as screen time than when defined as overall time spent being sedentary. More initiatives and guidelines combining all 3 movement behaviors will provide benefit with regard to adiposity, cardiometabolic risk factors, cardiorespiratory fitness, muscular physical fitness, well-being, health-related quality of life, mental health, academic performance, and cognitive/executive function.

## Abbreviations

ERIC: Education Resources Information Center
STROBE: Strengthening the Reporting of Observational Studies in Epidemiology

Child and adolescent movement behaviors (i.e., physical activity, sedentary behavior, and sleep duration) are individually associated with many similar physical (e.g., adiposity) (1–6), psychological (e.g., mental health) (7–9), and educational (e.g., academic performance) (3, 4, 9–14) outcomes. Spending time in one movement behavior displaces time spent in others, which may explain the overlap in associations with outcomes. However, these overlaps make it difficult to disentangle which behaviors are associated with specific outcomes. Therefore, researchers have started studying combinations of movement behaviors instead of studying them in isolation.

A previous systematic review by Saunders et al. (15) included 14 studies from 2011–2015 and found that the combinations of 1) high physical activity with low sedentary behavior, 2) high physical activity with high sleep duration, and 3) high physical activity, low sedentary behavior, and high sleep duration were associated with the most desirable physical health outcomes. This review informed several international health recommendations (16–19), including the Canadian 24-Hour Movement Behavior Guidelines for Children and Youth (20). Due to the popularity of these recommendations and the introduction of new analytical methods, such as compositional data analysis (21), research on movement behaviors has increased substantially. Therefore, an update to the previous review is needed.

While the associations between combinations of movement behaviors and physical health outcomes are known, many psychological and educational outcomes have yet to be systematically reviewed. With rising rates of depression and anxiety among children and adolescents (22, 23), psychological and educational outcomes are a growing concern, and thus research on these outcomes should be synthesized. Reporting of associations for combinations of movement behaviors with psychological and educational outcomes may inspire a wider range of professionals (e.g., teachers, clinical psychologists) to adopt interventions addressing multiple movement behaviors. In turn, these professionals may provide valuable input to alter guidelines to suit a broader range of outcomes. Therefore, we aimed to update and expand on the Saunders et al. review (15).

## METHODS

### Search strategy and selection criteria

We prospectively registered this systematic review on PROSPERO (https://www.crd.york.ac.uk/PROSPERO/; identification number: CRD42020181097), and we report our findings in line with the Preferred Reporting Items for Systematic Reviews and Meta-Analyses (PRISMA) guidelines (24).

To be included, studies needed to quantitatively analyze the association of at least 2 movement behaviors (i.e., physical activity, sedentary behavior, and sleep duration) with any outcome (i.e., physical, psychological, or educational) in youth (mean age 5–17 years). We modified the exclusion criteria from the previous review by not excluding studies based on sample size or type of physical activity measurement, whereas Saunders et al. (15) required a minimum sample size of 300. We placed no exclusion criteria based on study design, setting, publication status, or publication date. We included studies published in any language, provided they had an abstract in English with quantitative results.

We searched the following electronic databases in June 2020: MEDLINE (National Library of Medicine, Bethesda, Maryland), Cumulative Index to Nursing and Allied Health Literature (CINAHL) (EBSCO Industries, Birmingham, Alabama), PsychInfo (American Psychological Association, Washington, DC), SPORTDiscus (EBSCO Industries), PubMed (National Library of Medicine), Excerpta Medica Database (EMBASE) (Elsevier BV, Amsterdam, the Netherlands), and Education Resources Information Center (ERIC) (EBSCO Industries). We included more databases than the previous review, such as ERIC, to capture studies exploring a wider range of outcomes. Our search strategy can be found in Web Appendix 1 (available at https://doi.org/10.1093/aje/kwac212). We revised the previous search strategy to include the combination of any 2 movement behaviors and to cater the search strategy to more databases.

### Study selection, data extraction, and quality assessment

We uploaded all relevant articles to Covidence review management software (Covidence, Melbourne, Victoria, Australia (www.covidence.org)) and removed duplicates. Two independent reviewers screened titles and abstracts. Two reviewers independently screened full-text articles for studies that passed title/abstract screening. We resolved conflicts by consensus. One reviewer conducted bidirectional screening using the 14 articles from the Saunders et al. review (15) and 1 additional recent article. Bidirectional screening is a method wherein a reviewer screens all references within an article and any articles that cited the article (25), providing a more thorough literature search.

Two independent reviewers completed data extraction and quality assessment. Data items included the name of the lead author, the publication date, the sample size, the combination of movement behaviors evaluated, the outcomes measured, the measurement methods used, and the results. Preliminary searches and the Saunders et al. review (15) indicated that mainly observational studies would be included. Therefore, we evaluated study quality using an adapted version of the Strengthening the Reporting of Observational Studies in Epidemiology (STROBE) Checklist (26). As per previous reviews (27, 28), we rated studies on 9 criteria derived from STROBE items, since the absence of these items could potentially introduce bias. Studies were considered high-quality if they met 7 or more criteria.

### Data synthesis

There was substantial methodological heterogeneity across the studies, preventing a meta-analysis. Specifically, movement behaviors were categorized inconsistently. For example, some studies dichotomized movement behaviors (e.g., meeting/not meeting guidelines), others used sample-specific median splits, and others used compositional data analysis. While some studies could have been meta-analyzed (e.g., only those with isotemporal substitution), most would have been excluded, risking systematically biased results. Accordingly, we narratively compared combinations of movement behaviors relative to other combinations within the same study. Per the Cochrane Collaboration (29), comparisons were based on the direction of associations, not statistical significance. Exemplar studies were characterized to obtain potential associations.

Sedentary behavior was defined differently across studies (e.g., “screen time,” “sitting”). In this review, we have generally used the term “sedentary behavior.” We used “screen time” when we synthesized studies that all used “screen time” as their original definition of sedentary behavior. We noted instances where the definition of sedentary behavior influenced the results.

Due to the high number of studies that investigated different combinations of meeting the Canadian 24-Hour Movement Behavior Guidelines for Children and Youth (see Web Appendix 2), we refer to these recommendations as “movement behavior guidelines” in our synthesis. We synthesized data separately for children (mean age 5–13 years) and adolescents (mean age 14–17 years). These age ranges were chosen on the basis of current guidelines that separate sleep recommendations for children and adolescents (17, 20). We also analyzed whether objective versus subjective measures of physical activity influenced the results. Finally, “sleep” refers to sleep duration.

Table 1 provides a concise summary of our results. This table highlights general trends in the included studies on the basis of age group, outcome, and whether the studies investigated the combination of physical activity and sedentary behavior exclusively or whether the combination of all 3 movement behaviors was included. The combination of physical activity and sedentary behavior was addressed in the table because studies evaluating this combination made up a majority of the studies in this review.

**Table 1:** Associations of Combinations of Movement Behaviors (Physical Activity, Sedentary Behavior, and Sleep Duration) With Physical, Psychological, and Educational Outcomes in Children and Adolescents in a Systematic Review, 2002–2020

	**Combination of Movement Behaviors and Age Group** ^a^			
	**Physical Activity and Sedentary Behavior**		**Physical Activity, Sedentary Behavior, and Sleep Duration**	
**Outcome**	**Children**	**Adolescents**	**Children**	**Adolescents**
Adiposity	The combination of high physical activity and low sedentary behavior was most beneficial.	The combination of high physical activity and low sedentary behavior was most beneficial. Evidence suggested that sedentary behavior is more important during adolescence than in childhood.	The combination of high physical activity, low sedentary behavior, and high sleep level was most beneficial.	The combination of high physical activity, low sedentary behavior, and high sleep level was most beneficial.
Cardiometabolic risk factors	The combination of high physical activity and low sedentary behavior was most beneficial.	The combination of high physical activity and low sedentary behavior was most beneficial.	The combination of high physical activity and high sleep level was most beneficial.	No studies included sleep.
Cardiorespiratory fitness	A high level of physical activity was important, regardless of sedentary behavior.	The combination of high physical activity and low sedentary behavior was most beneficial.	The combination of high physical activity and high sleep level was most beneficial.	No studies included sleep.
Muscular physical fitness	The combination of high physical activity and low sedentary behavior was most beneficial.	No studies included adolescents.	The combination of high physical activity and high sleep level was most beneficial.	No studies included adolescents.
Well-being and socioemotional outcomes	The combination of high physical activity and low sedentary behavior was most beneficial.	The combination of high physical activity and low sedentary behavior was most beneficial.	The combination of high physical activity, low sedentary behavior, and high sleep level was most beneficial.	The combination of high physical activity, low sedentary behavior, and high sleep level was most beneficial.
Health-related quality of life	The combination of high physical activity and low sedentary behavior was most beneficial. Regarding sedentary behavior, screen time should particularly be addressed.	The combination of high physical activity and low sedentary behavior was most beneficial. Regarding sedentary behavior, screen time should particularly be addressed.	The combination of high physical activity, low sedentary behavior, and high sleep level was most beneficial.	No studies included sleep.
Mental ill health	The combination of high physical activity and low sedentary behavior was most beneficial.	The combination of high physical activity and low sedentary behavior was most beneficial.	The combination of high physical activity, low sedentary behavior, and high sleep level was most beneficial.	The combination of high physical activity, low sedentary behavior, and high sleep level was most beneficial.
Academic performance	The combination of high physical activity and high sedentary behavior was associated with positive academic performance. The combination of high physical activity and low screen time was associated with positive academic performance.	The combination of high physical activity and high sedentary behavior was associated with positive academic performance. The combination of high physical activity and low screen time was associated with positive academic performance.	The combination of high physical activity, low sedentary behavior, and high sleep level was most beneficial.	The combination of high physical activity, low sedentary behavior, and high sleep level was most beneficial.
Cognitive and executive function	The combination of high physical activity and low screen time was most beneficial.	No studies included adolescents.	The combination of low screen time and high sleep level was most beneficial.	No studies included sleep.

^a^ Children were defined as participants with a mean age of 5–13 years, and adolescents were defined as those with a mean age of 14–17 years.

## RESULTS

### Description of studies

**Figure 1:**
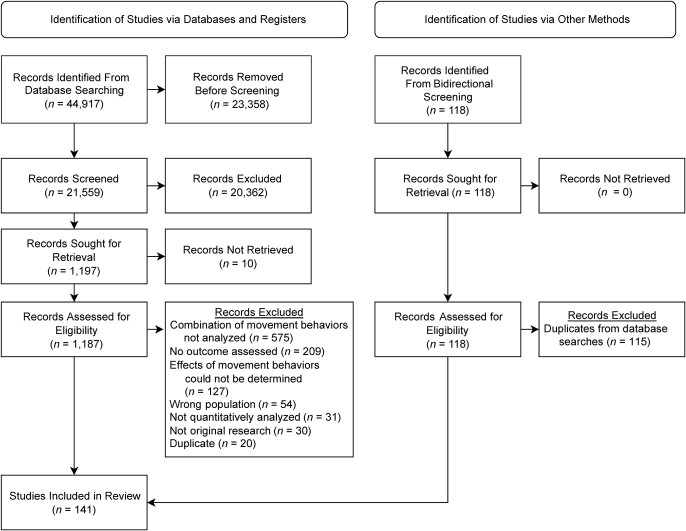
Selection of studies for inclusion in a systematic review of the associations of combinations of movement behaviors (physical activity, sedentary behavior, and sleep duration) with physical, psychological, and educational outcomes in children and adolescents, 2002–2020 followed the Preferred Reporting Items for Systematic Reviews and Meta-Analyses (PRISMA) guidelines (24).

We imported 44,917 references into Covidence. After removal of duplicates, 21,559 studies remained for title/abstract screening and 1,197 studies moved forward to full-text screening. Ten studies could not be retrieved after searching in academic libraries and requesting interlibrary loans. Three additional studies were added for full-text screening from the bidirectional screening process. Our total for data extraction included 141 studies (including 2 conference abstracts), all of which had an English version available (see Figure 1). A list of excluded studies can be found in Web Table 1.

Fifty-seven countries were represented across the studies, which were primarily from the United States (*n* = 29 studies), Canada (*n* = 21), and Australia (*n* = 19). Most studies (84%; 119/141) were high-quality (see Web Table 2) with a mean quality score of 7.4/9. The least reported items were “describe any efforts to address potential sources of bias” and “describe any methods used to examine subgroups and interactions.” Study characteristics can be found in Web Table 3.

Total physical activity, at any time of day or location, was primarily reported. Four studies reported on subdomains (e.g., sports). When studies were evaluated separately based on data being measured objectively or subjectively, the trends were the same. This was also true for study quality. The data set generated and analyzed during the current study is available in the Open Science Framework repository.

### Physical health–related outcomes

#### Adiposity.

Ten longitudinal studies and 79 cross-sectional studies investigated adiposity. Adiposity was measured by body mass index (weight (kg)/height (m)^2^), waist circumference, waist:height ratio, skinfold thickness, body fat percentage, visceral adipose tissue, and fat mass index (fat mass (kg)/height (m)^2^). Overall, participants with a higher level of physical activity and a lower level of sedentary behavior had less adiposity than persons with other combinations of these 2 behaviors. Results differed slightly by age group. For children, the most optimal combination was generally high physical activity and low sedentary behavior. For adolescents, a lower level of sedentary behavior was associated with lower adiposity, especially in girls. These results were consistent regardless of whether sedentary behavior was measured as electronic screen time or as total sedentary behavior. When sleep was analyzed, combinations with a high sleep level were associated with lower adiposity.

Ten longitudinal studies and 51 cross-sectional studies investigated the relationship between physical activity and sedentary behavior in adiposity. Investigators in the majority of those studies (6 longitudinal studies (29–34) and 32 cross-sectional studies (35–66)) concluded that the combination of high physical activity and low sedentary behavior was associated with the best adiposity outcomes among children and adolescents. For example, in one longitudinal study of 9,155 children and adolescents, youths with 5 bouts of moderate–vigorous physical activity per week and 4 hours of screen time per week had 25% lower odds of obesity 5 years later as compared with those with 3 bouts of moderate–vigorous physical activity and 25 hours of screen time per week (30). The remaining results differed by age (41–44, 47–49, 57–59, 61, 62, 68–76). Eleven studies used isotemporal substitution (2 longitudinal studies (77, 78) and 9 cross-sectional studies (44, 79–86)), all of which found that substituting sedentary behavior with moderate–vigorous physical activity was associated with lower adiposity.

Sixteen cross-sectional studies investigated the combinations of all 3 movement behaviors. All found that persons with a higher level of sleep had less adiposity than their peers (46, 66, 87–100). In 11 of these studies, the combination of high physical activity, low sedentary behavior, and high sleep had the best association with adiposity (46, 66, 87–93, 96, 97), while the remaining 5 studies had mixed results (90, 94, 95, 98, 99). An additional 12 cross-sectional studies used isotemporal substitution (77, 81, 86, 101–109) and found that substituting sedentary behavior with moderate–vigorous physical activity was associated with lower adiposity. There were mixed results on substituting sedentary behavior with sleep.

#### Cardiometabolic risk factors.

Three longitudinal studies and 18 cross-sectional studies evaluated cardiometabolic risk factors. Cardiometabolic risk factors assessed included systolic and diastolic blood pressure, insulin-related measures, triglycerides, cholesterol, and C-reactive protein. Overall, active children with a high sleep level had the most desirable levels of cardiometabolic risk factors. For adolescents, the combination of high physical activity and low sedentary behavior was associated with desirable levels of cardiometabolic risk factors.

Two longitudinal studies and 12 cross-sectional studies evaluated the association of combined physical activity and sedentary behavior with cardiometabolic risk factors. Researchers in all of the studies agreed that the combination of high physical activity and low sedentary behavior was associated with the best outcomes for cardiometabolic risk (48, 62, 64, 72, 74–76, 110–116). For example, investigators in one longitudinal study (*n* = 3,717) conducted a cluster analysis and found that persons with higher physical activity and lower sedentary behavior than their peers with lower physical activity but similar sedentary behavior had 13% lower odds of developing diabetes over a 5-year period (116). Investigators in 5 separate studies generally agreed that substituting sedentary behavior with moderate–vigorous activity was associated with the most desirable cardiometabolic outcomes (81, 85, 86, 108, 117). Researchers in one of these studies included sleep in their analysis and found mixed results, depending on the specific outcome, of substituting sedentary behavior with sleep (108). Findings from 2 studies (1 longitudinal (89) and 1 cross-sectional (118)), both in children, indicated that the addition of high sleep in combination with low screen time and/or physical activity yielded the most favorable cardiometabolic outcomes. No studies included sleep in adolescents.

#### Cardiorespiratory fitness.

One longitudinal study and 17 cross-sectional studies investigated cardiorespiratory fitness.

 Overall, combinations with high moderate–vigorous physical activity were associated with higher cardiorespiratory fitness. Specifically for adolescents, lower sedentary behavior was associated with higher cardiorespiratory fitness. Additionally, sleep was associated with higher cardiorespiratory fitness in children.

One longitudinal study in children (*n* = 315) found that substituting 30 minutes of sedentary behavior with vigorous-intensity physical activity yielded a positive association with cardiorespiratory fitness (β = 0.307) (119). Authors of 6 cross-sectional studies supported the longitudinal study’s finding by concluding that substituting sedentary behavior with moderate–vigorous physical activity was positively associated with cardiorespiratory fitness for children and adolescents (80, 82, 84, 102, 108, 120). An additional 6 cross-sectional studies found that children with higher physical activity than their peers, regardless of sedentary behavior, had higher cardiorespiratory fitness (37, 38, 121–124). However, in adolescents, 3 cross-sectional studies found that those who were more active and less sedentary than their peers had the highest cardiorespiratory fitness (47, 64, 125). Two studies that included all 3 movement behaviors found that children who were more active and slept longer than their peers had higher cardiorespiratory fitness (89, 126). No studies included sleep for adolescents.

#### Muscular fitness.

Three cross-sectional studies investigated children’s muscular fitness and found a positive association for those with high physical activity, low sedentary behavior, and high sleep duration.

Two cross-sectional studies determined that children’s spending more time in physical activity and less time in sedentary behavior was associated with better muscular fitness outcomes (70, 79). For example, a study in 2,506 children found those with at least 60 minutes of moderate–vigorous physical activity per day and low sedentary behavior (characterized by a median split) were 2.5 times more likely to fall into the “healthy zone” for flexibility than children with less than 60 minutes of moderate–vigorous physical activity per day and high sedentary behavior (70). The final study (*n* = 243) evaluated children meeting different combinations of movement behavior guidelines (126). Generally, children meeting physical activity and sleep recommendations had higher muscular strength, muscular endurance, and flexibility than children with any other combination of movement behaviors (126).

### Psychological outcomes

#### Well-being and socioemotional outcomes.

Two longitudinal studies and 10 cross-sectional studies investigated the association between well-being and socioemotional outcomes. The combination of high physical activity, low sedentary behavior, and high sleep had the most favorable outcomes with life satisfaction, happiness, stress, positive affect, negative affect, angriness, confusion, prosocial behavior, emotional health, peer problems, and hyperactivity.

Both longitudinal studies (127, 128) and 6 cross-sectional studies (129–134) found that children and adolescents who were more active and less sedentary than their peers had better socioemotional outcomes. For example, a longitudinal study carried out over 6 years in 3,979 children found that those who maintained low levels of physical activity and screen time or maintained physical activity levels but increased screen time had more socioemotional problems than those who increased their physical activity and maintained low levels of screen time (β = 0.46–0.74) (128). Four studies included sleep in their analysis. All found positive associations of the combination of high physical activity, low sedentary behavior, and high sleep with well-being (32, 89, 100, 108).

#### Health-related quality of life.

Three longitudinal studies and 6 cross-sectional studies investigated health-related quality of life. The combination of high physical activity and low sedentary behavior (particularly screen time) was associated with the best outcomes. The addition of sleep appeared to improve outcomes.

All studies investigating physical activity and sedentary behavior (3 longitudinal studies (127, 128, 135) and 5 cross-sectional studies (84, 136–139)) concluded that high physical activity and low sedentary behavior had the most positive association with health-related quality of life. The longitudinal study with the largest sample size (*n* = 3,979) found that children who maintained low levels of physical activity and screen time or maintained physical activity levels but increased screen time over 6 years had lower health-related quality of life than those who increased their physical activity and maintained low levels of screen time (β = −2.29–1.40) (128).

 In only 1 study did researchers include sleep in their analysis, and they found that those meeting all movement behavior guidelines had the best health-related quality of life (140).

#### Mental health.

Twelve cross-sectional studies examined depression and anxiety. Appropriate amounts of all 3 movement behaviors were associated with better mental health, but sleep appeared to have the most consistent positive associations.

Seven studies found that participants who were more active and less sedentary than their peers had better mental health (131, 141–146). All 5 studies that included sleep in their analysis suggested that high sleep may have a protective association with mental health, since combinations that included high sleep usually had the most desirable outcomes (142, 147–150). For example, a study in 20,078 adolescents found that those who met all movement behavior recommendations had the lowest odds of having anxiety or depression (147). However, combinations not including sleep were associated with the highest odds of having anxiety or depression (odds ratio = 3.92–37.14).

### Education-related outcomes

#### Academic performance.

One longitudinal study and 8 cross-sectional studies investigated academic performance. The association of high physical activity with low screen time or high physical activity with high total sedentary behavior was associated with the best academic performance for children and adolescents. Sufficient sleep seemed to be beneficial for academic performance.

Studies that measured sedentary behavior as screen time (1 longitudinal (151) and 2 cross-sectional (152, 153)) found that those who were more active with less screen time than their peers had the best academic performance. The longitudinal study (*n* = 261) found that children who participated in ≥60 minutes of moderate–vigorous physical activity per day and ≤2 hours of screen time per day were 2.75 times more likely to have better grades than children with less than 60 minutes of moderate–vigorous physical activity per day and more than 2 hours of screen time/day (151). However, children who engaged in more non–screen-based sedentary behavior (e.g., reading) had higher overall academic performance than their peers who spent less time in non–screen-based sedentary behavior, regardless of physical activity (67, 153–157). One study (*n* = 285) found that children with high physical activity and high total sedentary behavior had the best standardized test scores (504 vs. 471–502) (157). One study investigated the effect of combinations of all 3 movement behaviors on academic performance and found that meeting all movement behavior guidelines was the most beneficial (158). Furthermore, all combinations that included meeting the sleep recommendation were associated with higher academic performance than only meeting the combination of physical activity and screen time recommendations.

#### Cognitive/executive function.

Three cross-sectional studies examined associations of combinations of movement behaviors with cognitive and executive functioning in children. The combination of low screen time and long sleep duration was most beneficial.

One study found that children who were more active with less screen time than their peers had the highest level of executive functioning (159). Two additional studies included sleep in their analyses. Both studies found that children meeting the screen time and sleep guidelines had the most desirable associations with cognition and impulsivity as compared with all other combinations (160, 161). For example, the β coefficient for the effect of the association between low screen time and high sleep time on cognition was 3.21–5.37 (162).

### Additional outcomes

The remaining studies investigated nonalcoholic fatty liver disease, insomnia, and gross motor skills in children. They also studied the associations of vitamin D concentration, bone mineral content, hormone levels, and DNA methylation in adolescents with different combinations of physical activity, sedentary behavior, and sleep (113, 131, 163–167). There were only 1 or 2 studies for each of the above outcomes. Generally, researchers found that children and adolescents who were more active and less sedentary than their peers had the most favorable outcomes.

Although most studies found that those who were the most active, were the least sedentary, and slept the longest had the most optimal outcomes overall, we noted a few considerations based on outcome and age group. See Table 1 for a summary of the findings.

## DISCUSSION

In this systematic review, we aimed to improve our understanding of the associations of different combinations of physical activity, sedentary behavior, and sleep duration with physical, psychological, and educational outcomes in children and adolescents. Overall, we found that those who were more active, were less sedentary, and slept longer than their peers appeared to have the most favorable outcomes. Additionally, this review highlights the importance of sleep, both in practice and in future research. On several occasions, when only physical activity and sedentary behavior were considered in studies, we found that high physical activity and low sedentary behavior was the “best” combination. However, studies that included sleep found that combinations including high sleep levels were usually the superior option. For example, all combinations that included high sleep duration were associated with desirable mental health outcomes. Yet, out of 141 eligible studies, only 41 included sleep in their analyses. Fewer sleep studies were available for adolescents than for children. Possible explanations could be that researchers may study sleep more in children, since the onset of some disorders (e.g., attention-deficit/hyperactivity disorder) occurs at a younger age (168), or adolescent data were not available in the analyzed data sets (79, 126, 140, 160, 161).

Compromising on any movement behavior had consequences. A lower level of physical activity appeared to be associated with the most negative changes for physical health. Too much sedentary behavior, particularly screen time, was typically associated with poorer psychological health. Shorter sleep duration negatively affected all types of outcomes. For this reason, we cannot target one movement behavior without acknowledging the importance of the others. Including sleep in more longitudinal and intervention research could help investigators make stronger inferences about the associations of different combinations of movement behaviors with physical, psychological, and educational outcomes.

Limiting sedentary behavior appears to be especially important in adolescents. Low sedentary behavior in combination with high physical activity and/or sleep seemed to be crucial to achieving the most favorable adolescent physical health outcomes (e.g., adiposity). However, for children, studies found that low sedentary behavior did not appear to be important as long as physical activity and sleep levels were high. This may be explained by a potential association with total sitting time and poor dietary habits in adolescents (169). Additionally, the definition of sedentary behavior was important. For example, when sedentary behavior was defined as non–screen-based behavior, its association with academic performance was positive. This suggests that not all sedentary behavior is “bad.” Further, not all domains or types of physical activity may yield the same results. The most ideal combination for cognitive/executive functioning did not include high physical activity. Research shows that physical activity may be beneficial for cognitive development (170), but these associations may only be positive when accumulated through sports (171). Therefore, in future studies, researchers should consider exploring domain-specific movement behaviors and replicate findings on the less well-explored outcomes (e.g., executive function).

We synthesized data from 141 studies on 16 outcomes from 57 countries, making our review more reliable and generalizable than previous syntheses. However, limitations of our review should be considered when interpreting the data. First, we decided not to conduct a meta-analysis due to high heterogeneity in how the studies categorized, analyzed, and reported movement behaviors. For example, of the 3 studies on muscular fitness, 1 used isotemporal substitution, 1 considered whether or not children met all guidelines simultaneously, and 1 used sample-specific sedentary behavior median splits. As a result, we could not quantify the size of the pooled associations for the effect of each combination of movement behaviors on each outcome. More consistent reporting of associations across studies would facilitate meta-analyses. Alternatively, the wider availability of primary data would facilitate secondary analyses such as individual participant meta-analyses (172).

Finally, the included studies made some common methodological decisions that impaired our ability to draw firm conclusions. Many studies dichotomized movement behaviors using the current recommendations. This means that children who participated in 3 minutes of moderate–vigorous physical activity per day were analyzed the same way as children who participated in 59 minutes of moderate–vigorous physical activity per day, as both groups failed to meet the 60 minutes/day recommendation. Thus, the likely dose-response relationship for physical activity (and potentially other behaviors) could not be considered (1, 5, 173, 174). In future studies, investigators should explore isotemporal substitution or compositional data analysis, because these methods analyze the trade-offs in movement behaviors while accounting for dose-response associations. Further, the physical activity recommendation states that “muscle and bone strengthening activities should each be incorporated at least 3 days per week” (20, p. 319), but this was not included in any of the studies’ definition of meeting physical activity recommendations. Future research should account for this component of the guidelines, because resistance training has been shown to improve physical and mental health (175, 176).

In conclusion, the evidence suggests that physical activity, sedentary behavior, and sleep duration should be investigated in combination, not in isolation. Due to the consistent positive associations of sleep with a range of outcomes, we encourage researchers to consider sleep in their studies of movement behaviors. Guidelines, interventions, and public health campaigns should look beyond promoting single movement behaviors and move toward targeting all 3. The needs of children and adolescents could better be considered. Finally, children, adolescents, parents, and schools should be informed that physical activity, sedentary behavior, and sleep affect more than just children’s physical health; they also affect their psychological health and educational development.

## Supplementary Material

Web_Material_kwac212
