# Supplementary material for: Combinations of Physical Activity, Sedentary Behavior, and Sleep Duration and Their Associations With Physical, Psychological, and Educational Outcomes in Children and Adolescents: A Systematic Review
Source: Am J Epidemiol. 2022 Dec 14;192(4):665–79. doi: 10.1093/aje/kwac212 (PMC10089066; doi:10.1093/aje/kwac212)
Supplement: Web_Material_kwac212 [file web_material_kwac212.zip › kwac212 Sanders Web Material.zip!/kwac212 Sanders Web Material.pdf]

WEB MATERIAL

Combinations of Physical Activity, Sedentary Behavior, and Sleep Duration and Their Associations With Physical, Psychological, and Educational Outcomes in Children and Adolescents: A Systematic Review

Katrina Wilhite, Bridget Booker, Bo-Huei Huang, Devan Antczak, Lucy Corbett, Philip Parker, Michael Noetel, Chris Rissel, Chris Lonsdale, Borja del Pozo Cruz, and Taren Sanders

Contents

Web Appendix 1. Search Strategy by Database...1
Web Appendix 2. Canadian 24-Hour Movement Behavior Guidelines for Children and Youth..8
Web Table 1. Excluded Studies………………………………………………………….. (separate file)
Web Table 2. Quality Assessment………………………………………………….… (separate file)
Web Table 3. Study Characteristics……………………………………………….. (separate file)

WEB APPENDIX 1

Search Strategy by Database

MEDLINE:

S1 (MH “Exercise+”)
S2 (MH “Motor Activity+”)
S3 (MH “Physical Exertion+”)
S4 (MH “Sports+)
S5 TI/AB physic* N2 activ*
S6 TI/AB exercis* or sport*
S7 S1 or S2 or S3 or S4 or S5 or S6
S8 (MH “Sedentary Behavior+”)
S9 TI/AB sedentar*
S10 TI/AB low N2 “energy expenditure”
S11 TI/AB television or “screen time”
S12 TI/AB physical* N2 inactiv*
S13 S8 or S9 or S10 or S11 or S12
S14 (MH “Sleep+”)
S15 TI/AB sleep N2 duration
S16 TI/AB sleep
S17 S14 or S15 or S16
S18 (MH “Child+”)
S19 (MH “Adolescent+”)
S20 (MH “Students+”)
S21 TI/AB child* or adolescen* or “school age*” or boy* or girl* or student*
S22 S18 or S19 or S20 or S21
S23 S7 and S13 and S22
S24 S7 and S17 and S22
S25 S13 and S17 and S22
S26 S23 or S24 or S25

Filter: “all child: 0–18 years”

CINAHL:

S1 (MH "Exercise+")
S2 (MH "Motor Activity+")
S3 (MH "Physical Activity+")
S4 (MH "Sports+")
S5 TI/AB exercis* or sport*
S6 TI/AB physic* N2 activ*
S7 S1 or S2 or S3 or S4 or S5 or S6
S8 TI/AB sedentar*
S9 TI/AB “screen time” or television
S10 TI/AB low N2 “energy expenditure”
S11 TI/AB physical* N2 inactiv*
S12 S8 or S9 or S10 or S11
S13 (MH "Sleep+")
S14 TI/AB sleep N2 duration
S15 TI/AB sleep
S16 S13 or S14 or S15
S17 (MH “Child+”)
S18 (MH “Adolescence+”)
S19 (MH “Students+”)
S20 TI/AB child* or adolescen* or student* or “school age*” or boy* or girl*
S21 S17 or S18 or S19 or S20
S22 S7 and S12 and S21
S23 S7 and S16 and S21
S24 S12 and S16 and S21
S25 S22 or S23 or S24

Filter: “all child”

PsycInfo:

S1 (MJ Exercise)
S2 (MJ "Movement Therapy")
S3 (MJ "Physical Activity")
S4 (MJ "Sports")
S5 TI/AB sport* or exercis*
S6 TI/AB physic* N2 activ*
S7 S1 or S2 or S3 or S4 or S5 or S6
S8 (MJ “Sedentary Behavior”)
S9 TI/AB sedentar*
S10 TI/AB “screen time” or television
S11 TI/AB low N2 “energy expenditure”
S12 TI/AB physical* N2 inactiv*
S13 S8 or S9 or S10 or S11 or S12
S14 (MJ "Sleep")
S15 (MJ “Sleep Deprivation”)
S16 (MJ “Sleep Onset”)
S17 TI/AB sleep N2 duration
S18 TI/AB sleep
S19 S14 or S15 or S16 or S17 or S18
S20 (MJ “Child”)
S21 (MJ “Adolescent”)
S22 (MJ “Students”)
S23 TI/AB child* or adolescen* or student* or “school age*” or boy* or girl*
S24 S20 or S21 or S22 or S23
S25 S7 and S13 and S24
S26 S7 and S19 and S24
S27 S13 and S19 and S24
S28 S25 or S26 or S27

Filter: “childhood (birth–12 years)”, “adolescence (13–17 years)”

SPORTDiscus:

S1 (SU "Exercise")
S2 (SU "Physical Activity")
S3 (SU "Sports")
S4 TI/AB sport* or exercis*
S5 TI/AB physic* N2 activ*
S6 S1 or S2 or S3 or S4 or S5
S7 (SU “Sedentary Behavior”)
S8 TI/AB sedentar*
S9 TI/AB “screen time” or television
S10 TI/AB low N2 “energy expenditure”
S11 TI/AB physical* N2 inactiv*
S12 S7 or S8 or S9 or S10 or S11
S13 (SU "Sleep")
S14 (SU "Sleep Deprivation")
S15 (SU “Sleep Stages”)
S16 (SU “Sleep Hygiene”)
S17 TI/AB sleep N2 duration
S18 TI/AB sleep
S19 S13 or S14 or S15 OR S16 or S17 or S18
S20 (SU “Child”)
S21 (SU “Adolescent”)
S22 (SU “Students”)
S23 TI/AB child* or adolescen* or student* or “school age*” or boy* or girl*
S24 S20 or S21 or S22 or S23
S25 S6 and S12 and S24
S26 S6 and S19 and S24
S27 S12 and S19 and S24
S28 S25 or S26 or S27

ERIC:

S1 (SU "Exercise")
S2 (SU "Physical Activity")
S3 (SU "Sports")
S4 TI/AB sport* or exercis*
S5 TI/AB physic* N2 activ*
S6 S1 or S2 or S3 or S4 or S5
S7 TI/AB sedentar*
S8 TI/AB “screen time” or television
S9 TI/AB low N2 “energy expenditure”
S10 TI/AB physical* N2 inactiv*
S11 S7 or S8 or S9 or S10
S12 (SU "Sleep")
S13 TI/AB sleep N2 duration
S14 TI/AB sleep
S15 S12 or S13 or S14
S16 (SU “Child”)
S17 (SU “Adolescents”)
S18 (SU “Students”)
S19 TI/AB child* or adolescen* or student* or “school age*” or boy* or girl*
S20 S16 or S17 or S18 or S19
S21 S6 and S11 and S20
S22 S6 and S15 and S20
S23 S11 and S15 and S20
S24 S21 or S22 or S23

EMBASE:

S1 (SH "Exercise")
S2 (SH "Motor Activity")
S3 (SH "Physical Activity")
S4 (SH "Sports")
S5 TI/AB sport* or exercis*
S6 TI/AB “physical activity” or “physically active”
S7 S1 or S2 or S3 or S4 or S5 or S6
S8 (SH “Sedentary Behavior”)
S9 TI/AB sedentar*
S10 TI/AB “screen time” or television
S11 TI/AB “low energy expenditure”
S12 TI/AB “physically inactive” or “physical inactivity”
S13 S8 or S9 or S10 or S11 or S12
S14 (SH "Sleep")
S15 (SH "Sleep Deprivation")
S16 (SH “Sleep Latency”)
S17 (SH “Sleep Hygiene”)
S18 TI/AB sleep
S19 S14 or S15 or S16 or S17 or S18
S20 (SH “Child”)
S21 (MH “Adolescent”)
S22 (MH “Students”)
S23 TI/AB child* or adolescen* or student* or “school age*” or boy* or girl*
S24 S20 or S21 or S22 or S23
S25 S7 and S13 and S24
S26 S7 and S19 and S24
S27 S13 and S19 and S24
S28 S25 or S26 or S27

PubMed:

S1 (MH "Exercise")
S2 (MH "Motor Activity")
S3 (MH "Physical Exertion")
S4 (MH "Sports")
S5 TI/AB sport* or exercis*
S6 TI/AB “physically active” or “physical activity”
S7 S1 or S2 or S3 or S4 or S5 or S6
S8 (MH “Sedentary Behavior”)
S9 TI/AB sedentar*
S10 TI/AB “screen time” or television
S11 TI/AB “low energy expenditure”
S12 TI/AB “physically inactive” or “physical inactivity”
S13 S8 or S9 or S10 or S11 or S12
S14 (MH "Sleep")
S15 (MH "Sleep Deprivation")
S16 (MH “Sleep Stages”)
S17 (MH “Sleep Latency”)
S18 (MH “Sleep Hygiene”)
S19 TI/AB sleep
S20 S14 or S15 or S16 or S17 or S18 or S19
S21 (MH “Child”)
S22 (MH “Adolescent”)
S23 (MH “Students”)
S24 TI/AB child* or adolescen* or student* or “school age*” or boy or girl
S25 S21 or S22 or S23 or S24
S26 S7 and S13 and S25
S27 S7 and S20 and S25
S28 S13 and S20 and S25
S29 S26 or S27 or S28

Filter: “child: birth–18 years”

WEB APPENDIX 2

Canadian 24-Hour Movement Behavior Guidelines for Children and Youth

A healthy 24 hours (1) includes:

- Uninterrupted 9 to 11 hours of sleep per night for those aged 5–13 years and 8 to 10 hours per night for those aged 14–17 years, with consistent bed and wake-up times.
- An accumulation of at least 60 minutes per day of moderate to vigorous physical activity involving a variety of aerobic activities. Vigorous physical activities and muscle and bone strengthening activities should each be incorporated at least 3 days per week.
- Several hours of a variety of structured and unstructured light physical activities.
- No more than 2 hours per day of recreational screen time.
- Limited sitting time for extended periods.

Reference

1. Tremblay MS, Carson V, Chaput J-P, et al. Canadian 24-Hour Movement Guidelines for Children and Youth: an integration of physical activity, sedentary behaviour, and sleep. *Appl Physiol Nutr Metab*. 2016;41(6 suppl 3):S311–S327.
